# An NLRP3-stimulatory adjuvant improves the immunogenicity of influenza virus vaccines in mice and non-human primates

**DOI:** 10.1128/mbio.02343-25

**Published:** 2025-09-08

**Authors:** Kelsey Finn, Jonathan Chow, Dania Zhivaki, Debrup Sengupta, Holly Concepcion, Veronica Komoroski, Carolyn MacFarlane, Philip D. Coblentz, Milap Chokshi, Stephan Matissek, Emily Gosselin, Dahlia Alkekhia, Anastasia Nikiforov, Nicolina Lamberti, Victory Iheanyichukwu, Colin Kelly, Chisom Arinze, Andrew Cornforth, Sivan Elloul, Jonathan C. Kagan

**Affiliations:** 1 Corner Therapeutics 688067 , Watertown, Massachusetts, USA; Tsinghua University, Beijing, China

**Keywords:** immunization, influenza vaccines, innate immunity, dendritic cells, adjuvants

## Abstract

Dendritic cells (DCs) are the primary inducers of immunity induced by infection or vaccination. To stimulate durable T cell-mediated immunity, multiple DC activities are required. DCs must present antigen, express costimulatory molecules, and secrete inflammatory cytokines to direct T cell activation. These activities must be coordinated with DC migration to the lymph node and production of memory T cell-inducing cytokines, such as IL-1β. Common vaccination approaches use adjuvants that stimulate a subset of these activities, leading to diminished T cell activities and poor immune durability. We describe herein a lipid-based adjuvant, called a hyperactivator, which elicits all five of the aforementioned DC activities. Other adjuvants examined, including those used clinically, were defective for one or more of the key activities needed for robust T cell activation. Vaccines in mice and nonhuman primates targeting influenza virus antigens, including the quadrivalent commercial product Afluria, displayed enhanced adaptive immune responses when administered using hyperactivators as adjuvants. These data highlight the impact of hyperactivator adjuvants as a means to enhance antigen-specific immunity, with potential applications towards a universal influenza vaccine.

**IMPORTANCE:**

The generation of vaccines that stimulate T cell activities is an unmet need for the scientific community, as T cells are the primary mediators of immune memory. In this study, we report a new vaccine adjuvant called a hyperactivator. This hyperactivator adjuvant diversifies the T cell and antibody responses to virus antigens, such that in nonhuman primates, all influenza strains in the clinical vaccine Afluria are targeted. Hyperactivator adjuvants may represent a means to achieve the long-sought-after goal of a universal influenza vaccine.

## INTRODUCTION

Vaccines that prevent infectious diseases have saved over 150 million lives since 1974 (1), yet humanity continues to be at risk for infection. Several approaches to vaccination are in use clinically, including those consisting of live vaccines, inactivated pathogens, recombinant antigen subunits, and mRNA-encoded antigens (2). Regardless of the strategy used to generate protective immunity through vaccination, the process requires the engagement of dendritic cells (DCs). DCs are endowed with several activities needed for T and B cell-mediated adaptive immunity (3, 4). There are five key activities in DCs that are needed to stimulate new and long-lived antigen-specific lymphocyte responses (5). These activities include (i) major histocompatibility complex (MHC)-mediated presentation of antigenic peptides; (ii) T cell costimulatory molecule expression; (iii) effector T cell activating cytokine expression; (iv) DC migration to the lymph node that drains the immunized tissue; (v) production and release of cytokines that induce T cell memory, such as interleukin-1β (IL-1β). DCs lacking any of the five aforementioned activities are ineffective inducers of robust and durable anti-infective immunity. The upregulation of the five DC activities needed for long-term T cell activation depends on the receptors of the innate immune system. As such, vaccines commonly consist of antigens and adjuvants, the latter of which are chemical entities that activate innate immune signaling events (2). Examples of clinical-grade adjuvants include chemical agonists of the Toll-like Receptor (TLR) (e.g., monophosphoryl lipid A) or inflammasome pathways (aluminum hydroxide) (6, 7).

Recent studies have demonstrated that not all innate immune pathways are equally capable of inducing the activities in DCs needed for instruction of adaptive immunity. For example, TLR signaling in DCs is a poor inducer of bioactive IL-1β production and DC migration (8, 9). Despite this lack of IL-1β and migratory activities, the robust induction of several cytokines, antigen presentation, and costimulatory molecule expression by TLR ligands has led to the common description of these cells as “activated” (4, 10). An explanation for the inability of TLRs to maximally stimulate DC activities is derived from the recent appreciation that DCs do not only detect microbes and their products (via TLRs). DCs also detect tissue injury through the recognition of oxidized lipids (8, 11–13). Whereas detection of either oxidized lipid or microbial TLR ligands alone is not sufficient to activate all of the key DC activities needed for adaptive immunity, coincident detection of TLR ligands and oxidized lipids is capable of eliciting all five of these DC activities. As such, DCs exposed to TLR ligands + oxidized lipids are not considered to be active, but rather hyperactive (8, 11–13).

To date, studies of the hyperactive DC state (hDC) have focused primarily on the use of bacterial lipopolysaccharide (LPS) as a TLR ligand and mammalian oxidized lipids. Oxidized lipids of note include the heterogenous mixture oxPAPC (oxidized 1-palmitoyl-2-arachidonoyl-sn-glycero-3-phosphocholine) or a single pure component of oxPAPC called PGPC (1-palmitoyl-2-glutaryl-sn-glycero-3-phosphocholine) (8, 11–13). In murine models of vaccination, LPS +PGPC induces abundant populations of antigen-specific T cells that can infiltrate and eradicate implanted tumors (8, 9). Vaccinations with antigen plus LPS, antigen plus PGPC, or antigen plus aluminum hydroxide are less effective inducers of antitumor T cells. Such findings raise the possibility that stimuli of hDCs would be effective adjuvants for vaccination. However, neither LPS nor the oxidized lipids described to date are ideal tools to be considered for clinical development. LPS is recognized as too toxic for use in vaccines, and oxidized lipids are highly labile entities. Thus, alternative approaches to hyperactivate DCs would be useful to enable further exploration of hDC functions in immune defense. Herein, we describe a unique DC hyperactivator, consisting of the TLR7/8 ligand R848 and a synthetic lipid referred to as CT009. The combination of R848 and CT009 elicits the hDC state in murine and human systems and is capable of increasing the immunogenicity of the commercial influenza virus vaccine Afluria in mice and nonhuman primates. These collective findings support the importance of considering DC activation state in vaccine development and establish R848 + CT009 as a distinct type of adjuvant.

## RESULTS

### R848 and CT009 induce human moDC hyperactivation that potentiates T cell activation

**Fig 1:**
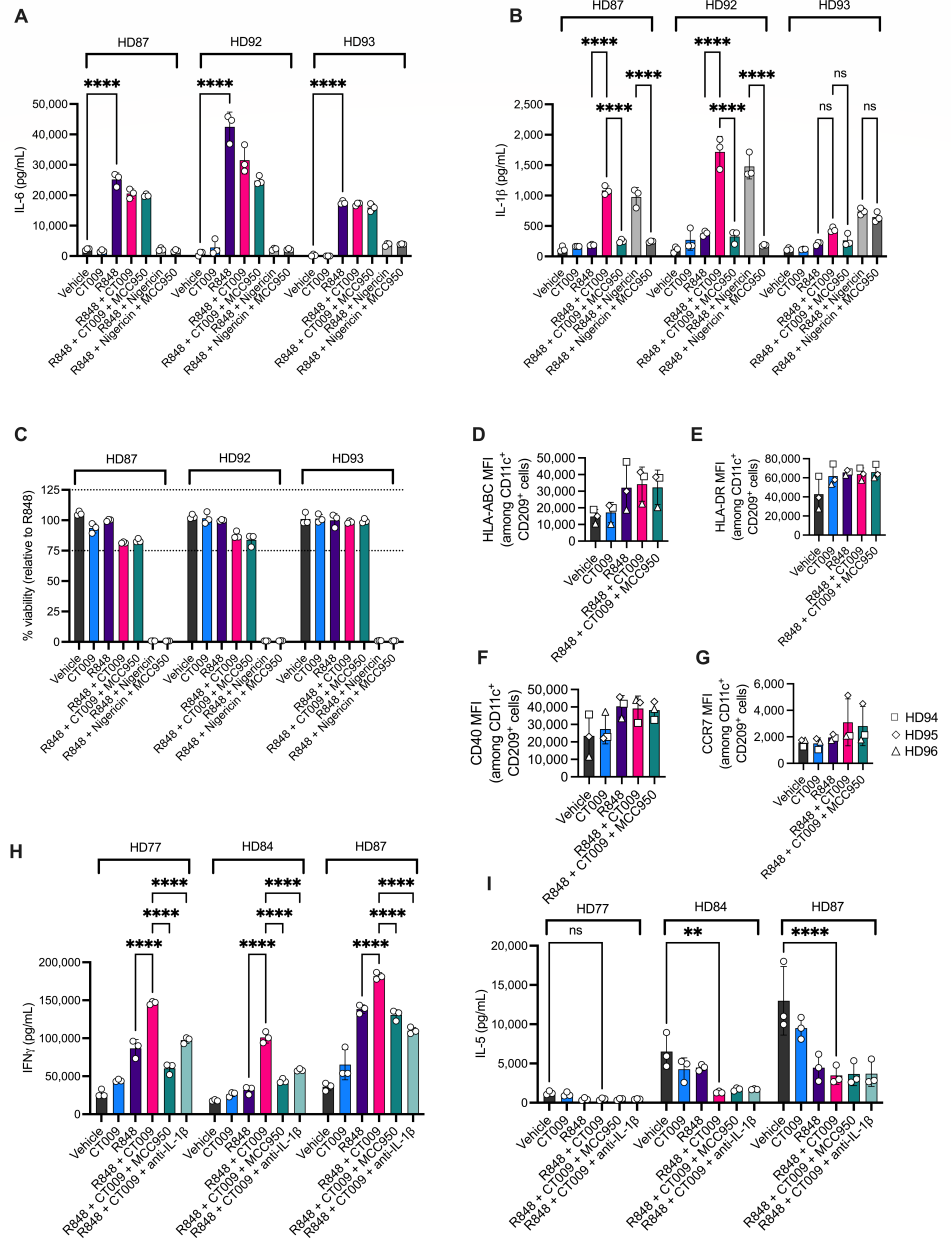
Hyperactivated human DCs promote a TH1 response. Human moDCs from three donors were treated for 1 day with 1 µg/mL R848 and/or 82.5 µM CT009. To compare responses to pyroptosis, cells were primed with R848 for 4 hours prior to nigericin (100 µM) treatment. In some test conditions, NLRP3 inhibitor MCC950 (10 µM) was added with R848. (**A**) IL-6 secretion and (**B**) IL-1β were measured from the cell culture supernatant. (**C**) CellTiter-Glo assay was used to measure relative cell viability. With another set of three donors, moDCs were treated with hyperactivating chemicals R848 and CT009. Cells were stained and analyzed by flow cytometry for median fluorescence intensity (MFI) of (**D**) HLA-ABC; (**E**) HLA-DR; (**F**) CD40; (**G**) CCR7. moDCs from three healthy donors (HD) were cocultured with autologous memory CD4 T cells in the presence of 1 µg/mL R848 and/or 41.3 µM CT009. Cultures utilized 0.1 ng/mL anti-CD3 soluble agonist antibody to activate T cells. Ten micromolar MCC950 and 10 µg/mL anti-IL-1β blocking antibody were used to test the dependency of T cell (**H**) IFN-γ and (**I**) IL-5 responses to IL-1β signaling. Each symbol represents one technical replicate (**A–C, H–I**) or mean value of technical triplicates for a single donor (**D–G**). Two-way ANOVA followed by Tukey’s multiple comparisons test was used to determine significant differences between treatment groups. **P < 0.01; ****P < 0.0001.

A library of lipids was screened for their ability to induce IL-1β from living, human monocyte-derived dendritic cells (moDCs) when combined with LPS. Work to develop a clinically suitable hyperactivation regimen identified the TLR7/8 agonist R848 to replace LPS. From the hits of the lipid screen, a novel derivative lipid CT009 was identified, the structure of which is illustrated in Fig. S1A. This lipid was selected due to its increased capacity to induce IL-1β release from living cells. Several batches of CT009 were manufactured, which had consistent particulate size, pH, and osmolality when formulated for use (Fig. S1B). The activities of R848 and CT009 were tested on moDCs. Treatment with R848 alone or in combination with CT009 induced the production of the NF-κB-dependent cytokine IL-6, whereas CT009 alone did not induce IL-6 (Fig. 1A). Neither R848 nor CT009 alone elicited production of IL-1β from moDCs; however, the combination of these stimuli induced IL-1β production (Fig. 1B). Treatment with MCC950, a small-molecule inhibitor of NLRP3 inflammasomes (14, 15), prevented the production of IL-1β induced by R848 + CT009-stimulated cells (Fig. 1B). As an additional comparison, moDCs were treated with R848 and a known NLRP3 agonist, nigericin (16). As expected, R848 + nigericin induced IL-1β production, which was inhibited by MCC950 (Fig. 1B). However, while treatment with R848 and CT009 in combination or as single agents had marginal effects on the viability of moDCs 1 day after stimulation, treatment with R848 + nigericin diminished the viability of cells (Fig. 1C). The observed reduction in cell viability after nigericin treatment correlated with the inability of moDCs to produce IL-6 (Fig. 1A). Similar to the results obtained with moDCs, R848 + CT009 induced IL-1β production from the monocytic human THP1 cell line in the absence of cell death (Fig. S1C and D). The combination of R848 and CT009 therefore induces the hallmark activity of cell hyperactivation, the NLRP3-dependent production of IL-1β from living human moDCs and THP-1 cells.

We next assessed the surface expression of markers important for DC function. MHC molecules are key mediators of antigen presentation, while CD40 is a costimulatory receptor that can enhance T cell activation. CCR7 is a chemokine receptor that is required for DC trafficking to lymph nodes. When moDCS from three different donors were treated with R848 and CT009, either as single agents or in combination, we observed no significant changes in the cell surface expression of MHC Class I (HLA-ABC), MHC Class II (HLA-DR), CD40, or CCR7 (Fig. 1D through G).

To determine if stimulated moDCs can enhance T cell responses, moDCs from three different donors were differentially stimulated and cultured with autologous memory CD4+ T cells. CD4+ T cells were activated using a low concentration of the agonistic CD3 antibody. In all donors examined, R848 + CT009 stimulations induced the highest amount of T cell activation, as assessed by IFNγ secretion (Fig. 1H). The increased IFNγ production from T cells was dependent upon IL-1β production from hDCs as both MCC950 treatment and IL-1β blocking antibodies reduced IFNγ to the levels observed with R848 treatments (Fig. 1H). The production of IFNγ was also dependent upon moDC-T cell interactions as treatment with R848, CT009, and anti-CD3 stimuli was insufficient to induce IFNγ when moDCs and T cells were cultured separately (Fig. S2A). IFNγ production was dependent upon anti-CD3 treatment (Fig. S2B).

**Fig 2:**
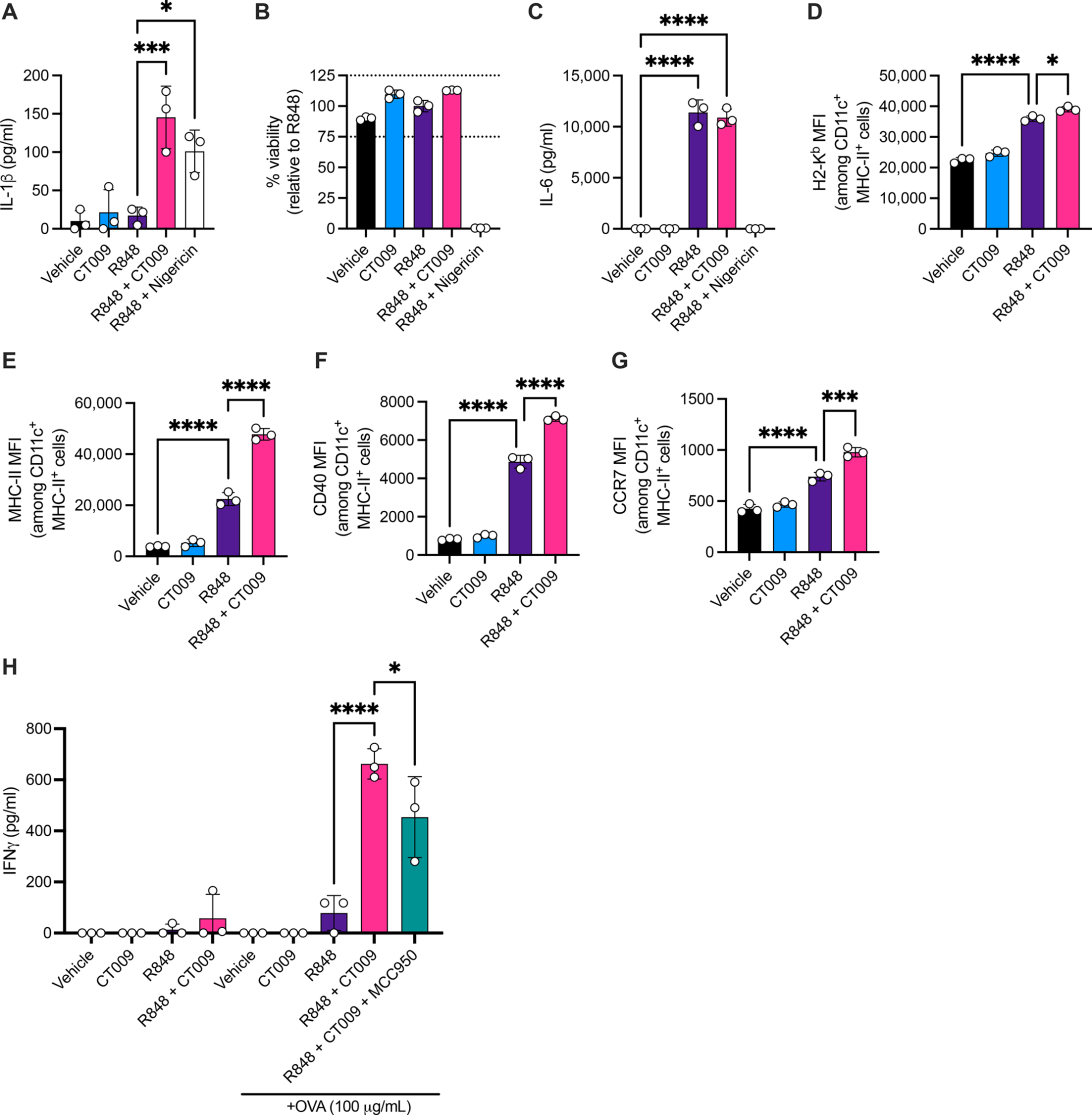
R848 and CT009 hyperactivate murine dendritic cells and promote T cell IFNγ responses. BMDCs were treated with 1 µg/mL R848 and/or 165 µM CT009 for 48 hours. Alternatively, cells were treated with R848 and 10 µM nigericin. (**A**) IL-1β secretion was measured by ELISA. (**B**) Relative cell viability was measured using CellTiter-Glo. (**C**) IL-6 was measured via ELISA. Treated cells were also stained at 48 hours to measure the MFI of (**D**) H2-K^b^ (MHC-I); (**E**) MHC-II; (**F**) CD40; (**G**) CCR7. (**H**) BMDCs were stimulated with various combinations of 1 µg/mL R848, 165 µM CT009, 100 µg/mL OVA, and 10 µM MCC950 for 16 hours, washed, and then plated at 2E4 cells/well. CD8 T cells isolated from spleens of OVA-immunized mice were cocultured with BMDCs at 2E5 cells/well. Four days later, IFNγ was measured from the cell culture supernatant. Means and SD are shown. Each symbol represents one technical replicate. One-way ANOVA followed by Tukey’s multiple comparisons test was used to identify statistically significant differences. *P < 0.05; ***P < 0.001; ****P < 0.0001.

In contrast to the results obtained with IFNγ, a marker for type 1 helper T cell (TH1) activities, R848 + CT009 suppressed the production of the TH2-associated cytokine IL-5 from T cells in two of three donors tested (Fig. 1I). These data support published findings using the hyperactivating combination of LPS + PGPC (8, 9), supporting the idea that hDCs induce TH1-biased T cell responses.

### R848 + CT009 hyperactivation of murine bone marrow-derived dendritic cells *in vitro* enhances the T cell response

To define the activities of R848 + CT009 in mice, we first determined if murine DCs were responsive to these hyperactivators *in vitro*. R848 + CT009 induced IL-1β production from murine bone marrow-derived DCs (BMDCs) with no evidence of viability defects (Fig. 2A and B). R848 + nigericin also induced IL-1β; however, this was associated with a loss of viability and an inability to produce IL-6 (Fig. 2A through C). Similar to observations in human moDCs, R848 or CT009 alone did not induce IL-1β production as compared to vehicle control, and R848 was required to stimulate IL-6 secretion (Fig. 2C). Treated BMDCs were also stained for MHC Class I (H2-K^b^), MHC Class II, CD40, and CCR7 expression. A clear activation phenotype was observed when cells were treated with R848, increasing the expression of these four markers compared to the vehicle treatment (Fig. 2D through G). In contrast to human hDCs, murine hDCs increased the expression of these molecules over cells treated with R848 alone (Fig. 2D through G).

To determine if murine hDCs could stimulate T cells, CD8+ T cells from mice immunized with chicken ovalbumin (OVA) were cocultured with BMDCs preincubated with combinations of OVA, R848, and CT009. Treatment with R848 + CT009 in the presence of antigen enhanced the IFNγ response from CD8+ T cells compared to R848 alone (Fig. 2H). Treatment with MCC950 reduced this IFNγ response.

### R848 + CT009 induces DC hypermigration

In addition to the ability to produce IL-1β while maintaining cell viability, hDCs display an enhanced ability to migrate (8). Migration enables DCs to deliver antigens from the periphery for presentation in T cell-rich lymph nodes. Lack of migratory ability has been a bottleneck to the success of DC-based therapeutics (17). To determine if R848 and CT009 induce hypermigratory behavior, *in vitro* and *in vivo* experimental approaches were taken.

**Fig 3:**
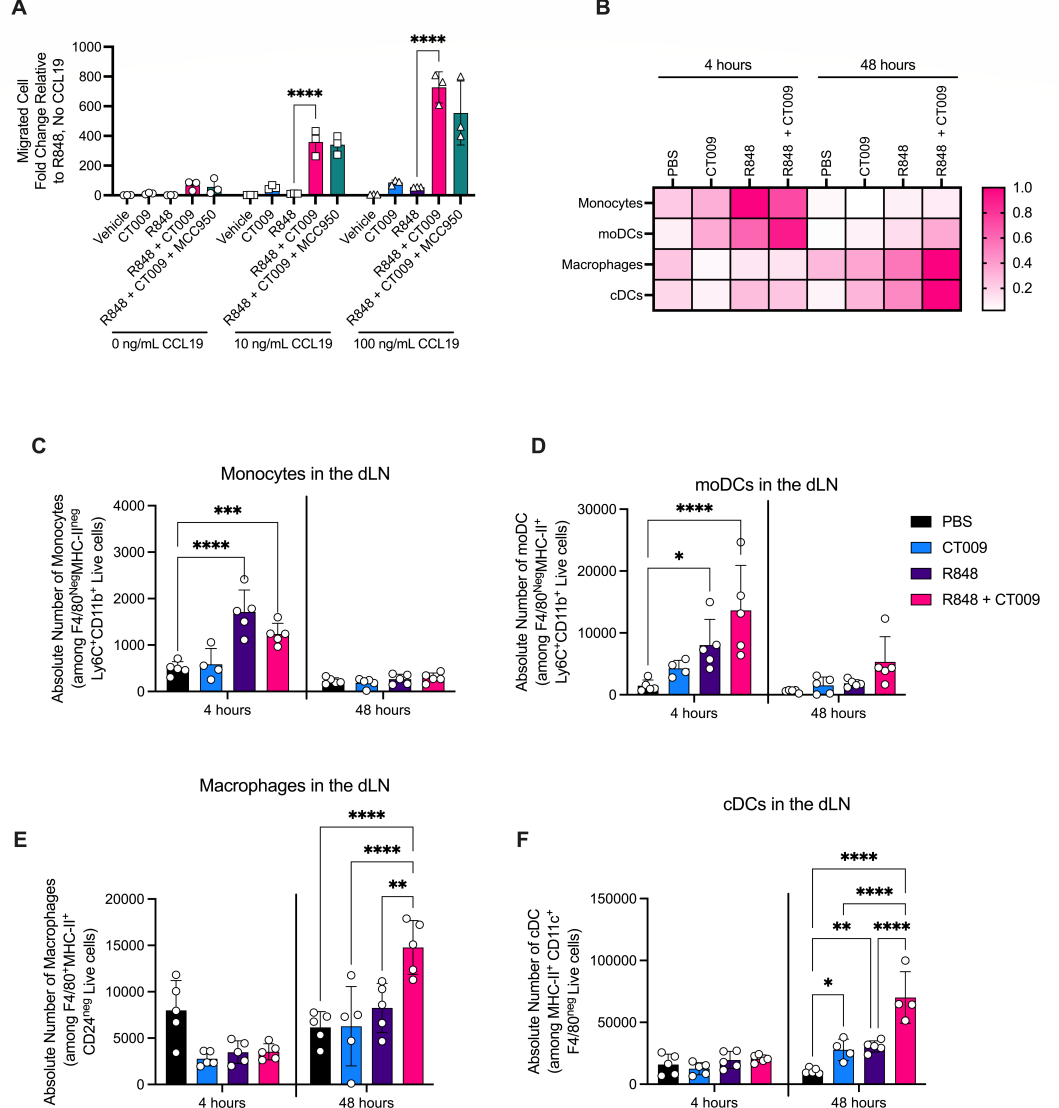
R848 and CT009 induce hypermigration of human and murine myeloid cells. (**A**) Human moDCs were treated overnight with 1 µg/mL R848 and 82.5 µM CT009 individually or in combination. Cells were collected and plated in the apical chamber of a transwell. Medium containing CCL19 was added to the basal chamber. After an overnight incubation, moDCs that migrated to the basal chamber were quantified. Means and SD are shown. Each symbol represents one technical replicate. Two-way ANOVA followed by Tukey’s multiple comparisons test was used to determine significant differences between treatment groups. ****P < 0.0001 (**B–F**) Fifty micrograms R848 and/or 100 µg CT009 were injected subcutaneously into C57BL/6J mice. Myeloid cells at the draining lymph nodes were quantified 4 hours and 48 hours post-injection. (**B**) Heat map shows the relative frequency of each cell type throughout the course of the study. Absolute counts enumerated by flow cytometry of (**C**) monocytes; (**D**) moDCs; (**E**) macrophages; (**F**) cDCs are also shown. Means and SD are shown. Each symbol represents one mouse. Two-way ANOVA followed by Sidak’s multiple comparisons test was used to determine statistical significance. *P < 0.05; **P < 0.01; ***P < 0.001; and ****P < 0.0001.

Human moDCs were treated with R848 and CT009 individually or in combination. After an overnight treatment, cells were placed in the apical chamber of a transwell filter plate. CCL19, the chemokine ligand for CCR7, was added to the media in the basal chamber to encourage cell migration. Without CCL19 addition, minimal moDC migration to the basal chamber was observed in any moDC treatment condition (Fig. 3A). In the presence of CCL19, R848 + CT009 treatments induced the highest frequency of moDCs detected in the basal chamber. CCL19-induced migration was dose-dependent as increasing the CCL19 concentration from 0 to 10 ng/mL to 100 ng/mL increased the number of cells that migrated to the basal chamber. Consistent with the NLRP3-independent nature of hDC hypermigration in mice (8), MCC950 treatment had no significant effect on CCL19-driven cell migration.

To test if R848 + CT009 have similar migratory effects on myeloid cells *in vivo*, mice were subcutaneously injected with these stimuli individually or in combination. Monocytes (F4/80−, MHC-II−, Ly6C+, CD11b+), moDCs (F4/80−, MHC-II+, Ly6C+, CD11b+), macrophages (F4/80+, MHC-II+, CD24−), and conventional DCs (cDCs) (F4/80−, MHC-II+, CD11c+) were quantified in the draining lymph nodes at 4 hours and 48 hours post-injection (Fig. 3B). At 4 hours post-treatment, R848 and R848 + CT009 induced an influx of monocytes and moDCs (Fig. 3C and D). At 48 hours, an increased number of macrophages and cDCs were detected in lymph nodes of mice treated with R848 + CT009 (Fig. 3E and F). R848 +CT009 drove the most robust increase in cDC migration as compared to other stimuli examined (Fig. 3F).

### R848 + CT009 is a potent vaccine adjuvant in mice

We determined the activities of hDC stimuli in the context of influenza vaccination. Afluria Quadrivalent (Afluria) is a commercially available influenza vaccine approved for clinical use, which contains hemagglutinin (HA) protein from four seasonal influenza strains. While influenza-specific antibodies play an important prophylactic role in neutralizing viral entry into cells, the T cell activities induced by vaccination have been less clearly defined (18). To understand the effects of R848 and CT009 on T cell and B cell responses, mice were immunized subcutaneously (s.c.) with Afluria alone, Afluria combined with R848, or Afluria combined with R848 + CT009. As an additional comparison, mice were immunized with Afluria combined with Alum, a canonical adjuvant that induces pyroptosis via the NLRP3 inflammasome pathway (19, 20). Mice were injected again on days 7 and 14, and antigen-specific T and B cells were assessed on day 21.

**Fig 4:**
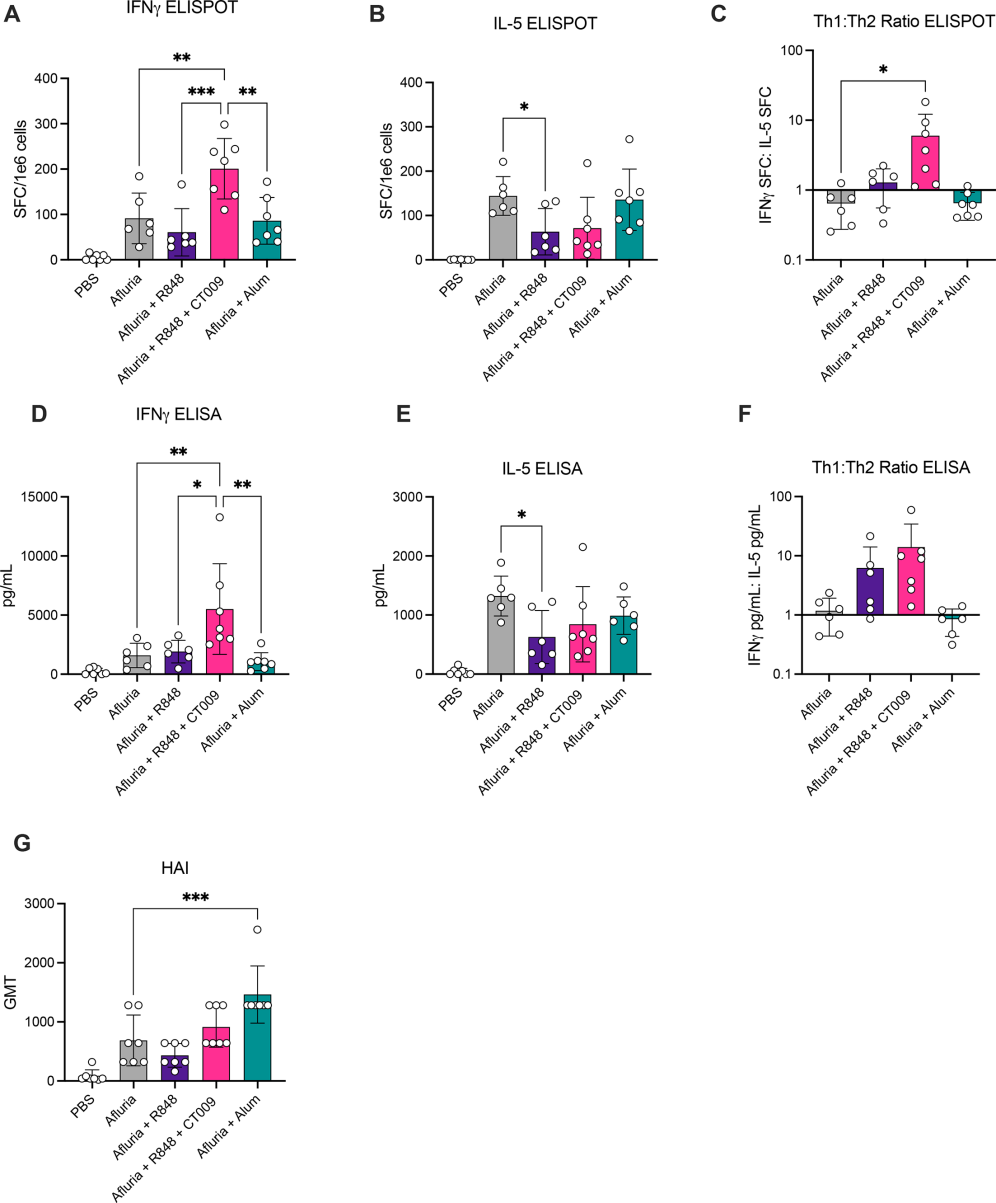
R848 and CT009 induce superior antigen-specific IFNγ T cell responses in mice when combined with Afluria vaccine. Splenocytes from immunized mice were stimulated with media alone or media containing Afluria. Antigen-specific T cells were quantified by IFNγ (**A**) or IL-5 (**B**) ELISpot. (**C**) The ratio of IFNγ+ SFCs to IL-5+ SFCs was calculated per mouse for assessment of Th1 vs Th2 skew. Total cytokine secretion from splenocytes restimulated with Afluria was measured via IFNγ (**D**) or IL-5 (**E**) ELISA. (**F**) The ratio of IFNγ to IL-5 secretion was calculated per mouse for assessment of Th1 vs Th2 skew. (**G**) Influenza-specific antibodies in serum were measured via HAI assay. Means and SD are shown. Each symbol represents one mouse; *n* = 6–7 mice per group. Significance was determined by one-way ANOVA Dunnett’s multiple comparisons to Afluria alone. *P < 0.05; **P < 0.01; ***P < 0.001.

Antigen-specific T cells in the spleen were quantified using Enzyme-linked ImmunoSpot (ELISpot). When restimulated with Afluria, splenocytes from mice immunized with Afluria showed increased numbers of IFNγ+ spot-forming cells (SFCs) compared to mice immunized with no antigen (PBS control). Notably, the induction of Afluria-specific IFNγ-producing T cells was maximal when R848 + CT009 was used as an adjuvant (Fig. 4A). Mice immunized with Afluria + R848 had similar IFNγ+ SFCs compared to mice immunized with Afluria alone (Fig. 4A), suggesting that TLR signaling alone was not sufficient to adjuvant the T cell response against influenza antigens in the Afluria vaccine. Similarly, Afluria combined with Alum did not increase the number of IFNγ-producing cells compared to Afluria alone (Fig. 4A).

In contrast to the robust induction of IFNγ-producing T cells by R848 + CT009, production of the TH2-associated cytokine IL-5 was most robust in vaccinations performed with non-adjuvanted Afluria or Afluria combined with Alum (Fig. 4B). By comparing the number of IFNγ SFCs to the number of IL-5 SFCs on a per mouse basis, we found that R848 + CT009 induced the strongest TH1 skew of all vaccination regimens examined (Fig. 4C). This finding may be of note, as type 1 immune responses driven by IFNγ-producing TH1 cells are important for viral clearance and protective immunity (21, 22).

In addition to quantifying the number of antigen-specific cells, the capacity for cytokine secretion was assessed by measuring IFNγ and IL-5 in cell culture supernatants after splenocytes were stimulated with Afluria for 72 hours (Fig. 4D and E). Similar to ELISpot data, Afluria combined with R848 + CT009 induced the highest level of IFNγ secretion (Fig. 4D). Comparison of IFNγ to IL-5 secretion on a per-mouse basis also demonstrated that R848 + CT009 induced the strongest TH1 skewed response (Fig. 4F).

To define the impact of R848 + CT009 on antibody responses induced by Afluria, HA-specific antibodies were assessed via the hemagglutination inhibition (HAI) assay. Mice immunized with Afluria combined with R848 + CT009 induced similar antibody titers compared to mice immunized with Afluria alone (Fig. 4G). This finding demonstrates that while R848 + CT009 mediate an adjuvant effect on T cells, this combination does not impact B cells in mice. Thus, in mice, R848 + CT009 induced the addition of T cell activities to an antibody response that can be elicited by antigens alone.

### Vaccines containing R848 + CT009 protect mice from lethal influenza virus infection

**Fig 5:**
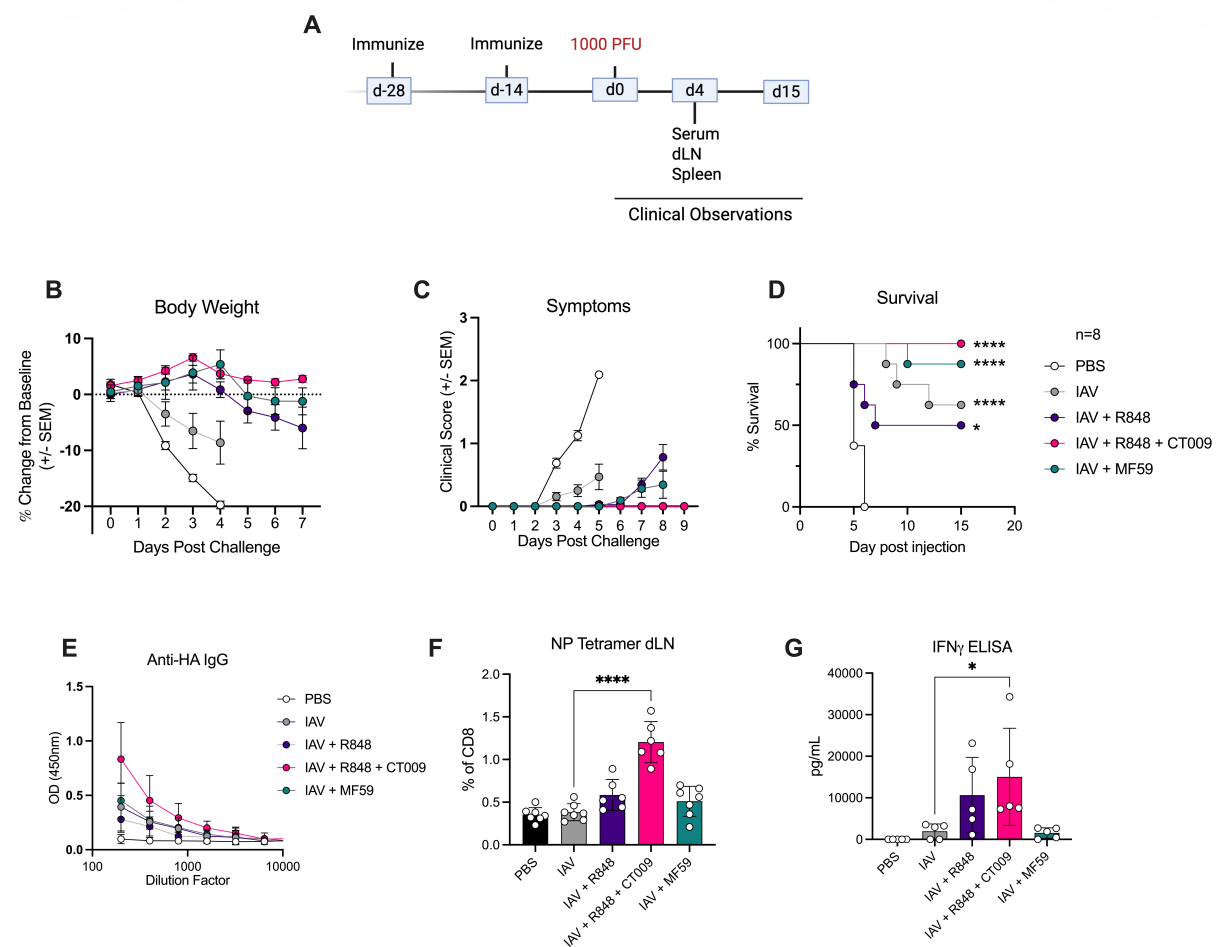
Including R848 and CT009 in vaccinations protects mice from lethal PR8 influenza challenge. (**A**) Timeline of study shows two vaccine doses preceding lethal dose infection of BALB/c mice with 1,000 plaque-forming unit (PFU) of PR8 virus. Mice were monitored for (**B**) body weight changes; (**C**) disease symptoms; (**D**) survival. For survival data, significance was determined compared to PBS. A cohort of mice was sacrificed on day 4 post-infection for assessment of (**E**) HA-specific antibodies; (**F**) NP-specific CD8 T cells in draining lymph nodes; (**G**) IFNγ secretion from splenocytes after restimulation with IAV. Means and SD are shown. Each symbol in the bar graph represents one mouse; *n* = 5–8 mice per group. Significance was determined by log-rank Mantel-Cox test (**D**), Two-way ANOVA followed by Dunnett’s multiple comparison (**E**) or one-way ANOVA followed by Tukey’s multiple comparisons (**F–G**). *P < 0.05; ****P < 0.0001.

To determine the impact of hDC stimuli in the context of infection, we used the PR8 mouse model of influenza. For these studies, heat-inactivated PR8 virus (IAV) was utilized as the source of antigen. Mice were immunized with IAV alone or combined with R848 or R848 + CT009. As an additional comparison, mice were immunized with IAV combined with MF59, a squalene-based adjuvant commonly used in influenza vaccines, particularly those targeted for the elderly population (23). Mice received two prophylactic injections spaced 2 weeks apart and were subsequently challenged with a lethal dose of PR8 virus (Fig. 5A). Mice were monitored for body weight changes, signs of disease, and survival. Signs of disease included assessments of hair coat, breathing, conjunctivitis, and motility. Based on these symptoms, mice were assigned a score with 0 representing no signs of illness and 3 representing mice with the worst symptoms. Additionally, immune responses were assessed in a cohort of mice 4 days after infection.

Mice vaccinated with PBS exhibited weight loss and signs of disease after infection, resulting in 100% lethality (Fig. 5B through D). All antigen-containing immunizations, even those lacking adjuvants, protected mice from viral disease and lethality (Fig. 5B through D). Similarly, at 4 days post-viral challenge, all adjuvants and antigen combinations induced comparable antibody responses in the serum (Fig. 5E). To define the impact of distinct adjuvants on T cell activities, cells were collected from the draining lymph node 4 days post-infection and stained to identify nucleoprotein (NP)-specific CD8+ T cells. Notably, the only immunization group demonstrating a significant NP-specific T cell expansion was the group vaccinated with R848 + CT009 (Fig. 5F). Usage of R848 or MF59 did not increase NP-specific CD8+ T cells, as compared to unvaccinated or IAV vaccinated groups (Fig. 5F). To further characterize T cell responses, splenocytes were restimulated with IAV for 72 hours, and the supernatant was collected to measure IFNγ production. Compared to IAV vaccination, addition of R848 + CT009 increased the IFNγ response, which did not occur with other adjuvants (Fig. 5G). These data suggest that the major impact of the R848 + CT009 adjuvant is to increase antigen-specific T cell responses to vaccination.

### In nonhuman primates, R848 + CT009 increased the immunogenicity of the FDA-approved influenza virus vaccine Afluria

**Fig 6:**
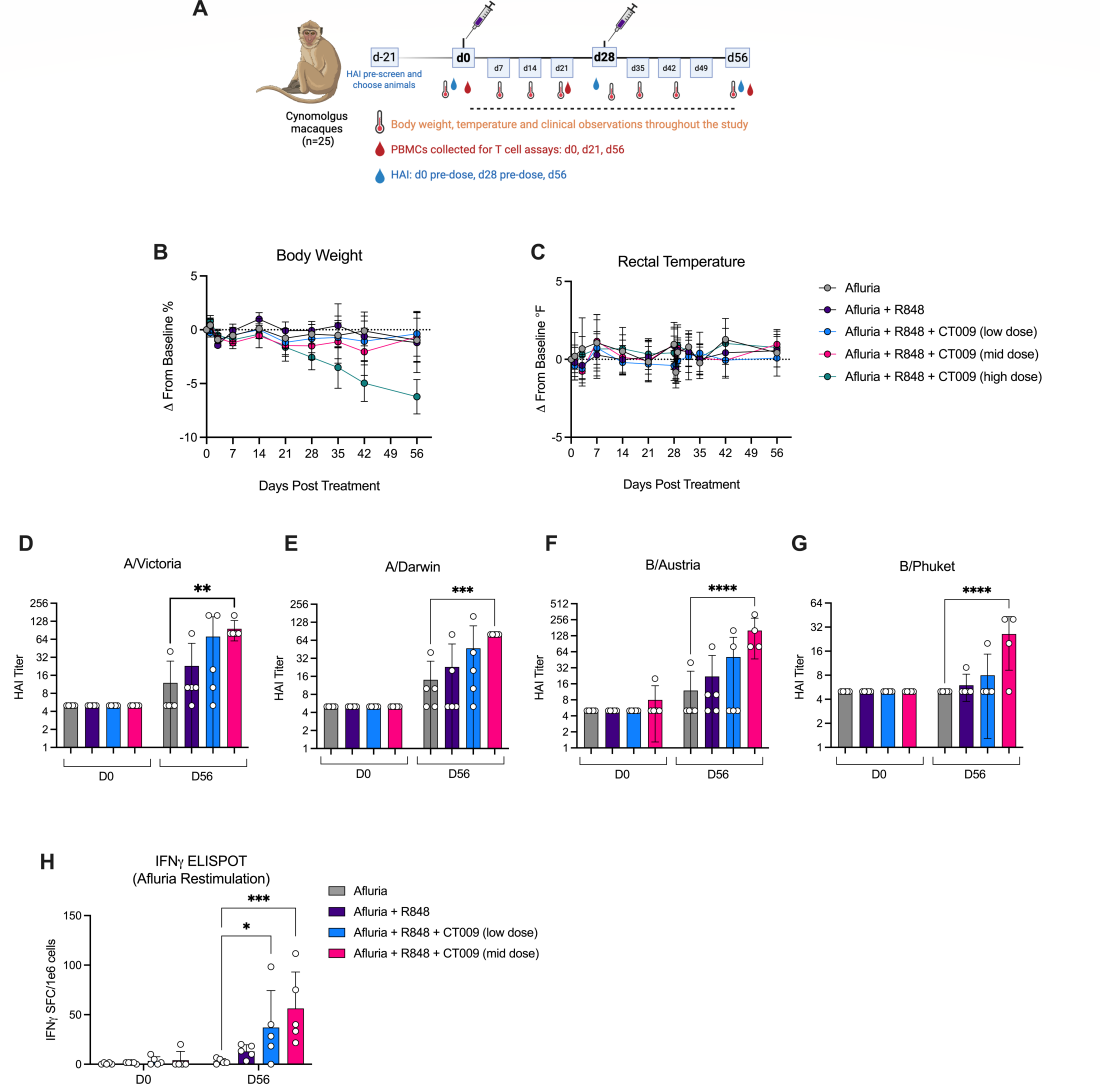
R848 and CT009 induce superior antibody and T cell responses in NHPs when combined with Afluria vaccine. (**A**) A total of 25 flu-naïve *Cynomolgus macaques* received two subcutaneous vaccinations spaced 4 weeks apart. Blood was collected at the indicated time points for T cell and antibody responses. (**B**) Body weight and (**C**) rectal temperature were measured weekly. (**D–G**) HAI titers against each influenza strain in Afluria were measured from serum on days 0 and 56. (**H**) Antigen-specific T cell responses were measured by IFNγ ELISpot from PBMCs taken on days 0 and 56. Reported responses have unstimulated background signals subtracted. Each symbol in bar graphs represents one animal. Means and SD are shown. Significance was determined by two-way ANOVA with Dunnett’s multiple comparisons test to Afluria. **P < 0.01; ***P < 0.001; ****P < 0.0001.

To determine if the ability of DC hyperactivators to operate as T cell stimulatory adjuvants extends beyond rodents, immunizations of nonhuman primates (NHPs) were performed. *Cynomolgus macaques* naïve to influenza infection or vaccination were chosen for the study based on negative HAI assay prescreen results. Animals were vaccinated with Afluria subcutaneously. Additional cohorts were vaccinated with Afluria combined with R848 alone or with various doses of R848 + CT009. NHPs were vaccinated twice, separated by 4 weeks (Fig. 6A). In this non-terminal study, blood was collected to monitor B and T cell activities prior to vaccinations and in the ensuing days after vaccination. Animals were monitored up to 56 days after the initial immunization.

Although the experiment was not designed to be a comprehensive toxicity study, body weight, temperature, and injection site reactivities were monitored. Most animals maintained their body weight throughout the length of the study, with the exception of animals receiving the highest dose of CT009 (Fig. 6B). Despite the loss in body weight with the high dose, none of the vaccinated primates exhibited meaningful changes in body temperature at any point in the study (Fig. 6C). The injection sites were also monitored for reactivity and scored for swelling (edema) and redness (erythema). Although animals were observed to have some edema and erythema, no clear trends tracked with dose of the injected materials (Fig. S3A and B). Based on these data, we concluded that R848 combined with the low and intermediate doses of CT009 was well tolerated and was most relevant in assessing immunological responses.

To compare antibody responses among the treatment groups, serum was collected on days 0, 28, and 56 and tested in HAI assays for reactivity against the four influenza virus strains included in Afluria. On day 28 after a single dose, antibodies generated against A/Victoria, A/Darwin, B/Austria, and B/Phuket were not increased by Afluria alone (Fig. S3C through F). In contrast, antibodies specific for these viral strains increased in groups containing CT009 in a dose-dependent manner (Fig. S3C through F). On day 56, 4 weeks after the second immunization, antibody titers were increased for all groups containing CT009, as compared to antibody titers prior to immunization (Fig. 6D through G). By day 56, NHPs that received the mid dose of CT009 had the highest antibody responses against all four flu strains (Fig. 6D through G).

To study the T cell response, peripheral blood mononuclear cells (PBMCs) collected on days 0, 21, and 56 were plated for IFNγ ELISpot and restimulated with Afluria. T cell responses required a second vaccination as no T cell responses were detected on day 21 (Fig. S3G). On day 56, minimal IFNγ responses were observed when animals were vaccinated with Afluria alone (Fig. 6H). The addition of R848 did not enhance the IFNγ signal above the background; however, the addition of R848 + CT009 increased the number of IFNγ-producing cells in a CT009 dose-dependent manner (Fig. 6H). Overall, these NHP studies demonstrate the superior B and T cell immunogenicity of hyperactivating adjuvants, in the context of the clinical influenza vaccine Afluria.

## DISCUSSION

Inducing potent and durable T cell responses through vaccination has been a challenging endeavor of the field. A recent example of this challenge was illustrated by the vaccines for SARS-CoV2. These vaccines induce robust antibody responses to viral antigens, but T cell responses are weak when comparing vaccine recipients to naturally infected individuals (24). T cells play a role that cannot be replaced by a B cell response, and in numerous diseases such as HIV infection and cancer, T cells may be key to disease clearance. Our finding that hyperactivator adjuvants consisting of R848 + CT009 increased T cell responses in mice and NHPs is therefore a notable point of distinction from adjuvants currently in clinical use.

Also of note, the T cell responses induced by R848 + CT009 were skewed toward a TH1 phenotype. In contrast, Alum, the most common adjuvant used for vaccines in the USA, skews responses toward a TH2 phenotype (25). Hyperactivators may therefore offer an adjuvant option that could skew the immune response toward a favored TH1 phenotype.

We found that the induction of a T cell response by hyperactivator adjuvants did not come at the cost of losing the B cell response. Indeed, hemagglutination inhibition remained the same when vaccinating mice with Afluria or Afluria in combination with R848 + CT009 (Fig. 4G). Interestingly, hemagglutination inhibition assays in NHPs demonstrated higher titers resulting from R848 + CT009 addition (Fig. 6D through G).

With the ability to mount both B and T cell responses in NHPs, hyperactivator adjuvants could enable mechanistic studies to determine the contribution of T cells to immunity during diverse infections. Moreover, the increased breadth of influenza antibodies generated in NHPs that received hyperactivator vaccines offers possibilities in the context of a broadly reactive universal influenza vaccine (26).

Although seasonal flu vaccines are formulated to target multiple strains, there is no guarantee that patients develop immunity against all the strains. In the NHP experiment, HAI titers against B/Phuket did not increase in animals receiving non-adjuvanted Afluria, which is the FDA-approved product in clinical use. In animals receiving increased doses of CT009 + R848, HAI titers against B/Phuket increased over the course of the study. One must keep in mind the caveat that there were only five animals per treatment group, but the data suggest that a tolerable dose such as the mid dose could improve immune responses while minimizing unwanted side effects.

The discoveries presented in this study suggest that R848 + CT009 act in concert to induce DC hyperactivation, ultimately leading to enhanced adaptive immunity. Hyperactivating adjuvant may therefore be useful to improve the efficacy and durability of current vaccines and for developing new ones. These findings provide a mandate to explore the value of lipid hyperactivators as vaccine adjuvants, in various contexts of infectious disease.

## MATERIALS AND METHODS

### Hyperactivator preparation and additional reagents

R848 was either purchased (Invivogen) or custom-manufactured at Aurigene Pharmaceutical Services in its salt form and dissolved in either physiological water or PBS. Resuspended stocks were frozen at −20°C or lower. CT009 (manufactured at Aurigene) was resuspended in a PBS solution containing 4% Kolliphor P407. Sizing of formulated CT009 particulates was done using the MasterSizer 3000 (Malvern). For *in vitro* studies, stock solutions were then further diluted in PBS before being added to cultures at the target concentration. As a negative control, vehicle conditions received an equivalent amount of PBS containing Kolliphor P407. For *in vivo* studies, stock CT009 was diluted with PBS when required. Nigericin and MCC950 (Invivogen) were prepared as directed by the manufacturer.

### THP1-Null2 cell activation studies

THP1-Null2 cells (InvivoGen) are a human monocytic cell line that produces IL-1β in response to treatment with R848 and CT009. THP1-Null2 cells were plated at 1E5/well in 96-well plates and treated with chemicals for 48 hours. Supernatants were then collected, and IL-1β secretion was measured using Lumit immunoassays (Promega). The viability was measured using CyQUANT LDH Cytotoxicity Assay (Invitrogen) according to the manufacturer’s instructions.

### Human moDC differentiation

Human cell samples were obtained from Miltenyi and AllCells. Collections were subject to Institutional Review Board (IRB) protocol approvals by vendors, and donors were virally screened at least every 90 days to be negative for human immunodeficiency virus, hepatitis B virus, and hepatitis C virus infections. Cell samples were de-identified for end users and not human subject research. Human monocytes were positively selected from fresh leukapheresis blood products using StraightFrom Leukopak CD14 Isolation Kits (Miltenyi) according to the manufacturer’s protocol. Cells if not used fresh were stored frozen in FBS containing 10% DMSO in a vapor phase liquid nitrogen freezer. Monocytes were differentiated into moDC by culturing in RPMI1640 containing 10% fetal bovine serum, penicillin/streptomycin, beta-mercaptoethanol, sodium pyruvate, L-glutamine, non-essential amino acids, and HEPES (R10++) for 6 days with 50 ng/mL GM-CSF and 25 ng/mL IL-4 recombinant cytokines (Miltenyi). Half the original volume of R10++ with 50 ng/mL GM-CSF and 25 ng/mL IL-4 was added to culture flasks on day 3 as a feed, and cells were used on day 6 for experiments.

### Human moDC cytokine secretion, viability, and activation marker staining

To study effects of R848 and CT009 on human moDC, cells were plated at 1E5 cells/well in 96-well plates and treated with chemicals for 24 hours. IL-6 and IL-1β secretion were measured using Lumit immunoassays (Promega). Viability was measured using CellTiter-Glo 2.0 (Promega) according to the manufacturer’s instructions.

When cells were stained to measure the expression of activation markers, cells were collected 1 day after chemical treatments and stained with live/dead viability dye in the dark at 4°C for 10 minutes (ThermoFisher). Samples were then treated with Fc receptor block (BD Biosciences) for an additional 10 minutes at 4°C. Cells were resuspended with a primary CCR7 antibody and incubated at 37°C for 30 minutes. moDCs were incubated with a biotinylated secondary antibody afterward at 4°C for 20 minutes. Finally, cells were resuspended in an antibody mix containing streptavidin-bound fluorophore and additional surface staining antibodies for incubation at 4°C for 20 minutes. Cells were fixed using 4% paraformaldehyde in PBS by incubating for 20 minutes in the dark at room temperature. After washing samples, cell staining was analyzed using a BD Biosciences FACS Symphony A3 flow cytometer.

### Human moDC coculture with memory CD4+ T cells

Autologous memory CD4+ T cells were isolated from the CD14-negative fraction of leukopaks when isolating monocytes. A custom PBMC isolation kit (Miltenyi) was used to deplete red blood cells and granulocytes. A memory CD4 T cell isolation kit (Miltenyi) was then used to negatively select for the desired cells. Cells were stained to confirm the purity to be at least 90% memory CD4 T cells by CD3, CD4, and CD45RO staining of live cells. Cells were stored for use by freezing in FBS containing 10% DMSO.

To coculture moDC and memory CD4 T cells, 50,000 moDCs were seeded into U-bottom 96-well plates with 250,000 memory CD4 T cells. Cells were treated with or without R848 and CT009 to stimulate moDCs. To activate T cells in culture, 0.1 ng/mL anti-CD3 was added. Anti-IL-1β blocking antibody (Biolegend) was used at 10 µg/mL. After 2 days of coculture, the cell culture supernatant was collected for cytokine analysis via IFNγ Lumit (Promega) and cytokine bead array LegendPlex kits (Biolegend).

### Mice

Female C57BL/6J or BALB/c mice aged 6–8 weeks were purchased from The Jackson Laboratory. Procedures and experimental group sizes were approved by the regulatory authorities for animal welfare. All mice were kept in accordance with federal and state policies on animal research at CRADL Vivarium facilities.

### Mouse FLT3L BMDC differentiation and chemical treatments

Bone marrow from femurs and tibia was collected from C57BL/6J mice and ACK lysed for 1 minute to remove red blood cells. Cells were filtered through a 40 µm cell strainer. After counting, cells were plated at 8E6 cells/well in a 12-well tissue culture plate in IMDM media containing 10% fetal bovine serum, penicillin/streptomycin, and L-glutamine (I10). Recombinant FLT3L (Miltenyi) was added to cultures at a final concentration of 200 ng/mL, and cells were allowed to differentiate for 9 days in a 37°C incubator. To hyperactivate BMDCs, cells were collected and treated at 2E5 cells/well in a 96-well plate in I10 media containing 50 ng/mL FLT3L. Cells and culture supernatant samples were collected 48 hours later.

### Mouse FLT3L BMDC coculture with CD8 T cells

Mice were immunized subcutaneously with 500 µg chicken ovalbumin (OVA) emulsified at a 1:1 volumetric ratio with incomplete Freund’s adjuvant (IFA). Seven and 14 days later, mice were boosted with 200 µg OVA. Thirty-five days after the initial immunization, spleens and lymph nodes were collected and mechanically dissociated into a single-cell suspension through 70 µm cell strainers. ACK lysis buffer was used to remove red blood cells, and cells were resuspended in MACS buffer (Miltenyi). CD8 microbeads (Miltenyi) were used to positively select for CD8 T cells. Cells were stained to assess the purity of isolation based on CD3 and CD8 expression.

FLT3L BMDCs were treated for 16 hours with or without R848, CT009, and OVA. Cells were then collected and washed by layering them on an FBS cushion and pelleting at 400 g for 5 minutes. Cells were washed twice more in I10 media and plated in round-bottom 96-well plates at 20,000 cells/well. CD8 T cells were plated at 200,000 cells/well. After 4 days, the cell culture supernatant was collected to quantify IFNγ secretion using IFNγ Lumit kits (Promega).

### Human *in vitro* transwell migration assay

After differentiation into moDCs, 4E6 cells were plated into 10 cm Petri dishes along with combinations of R848, CT009, and MCC950. Cells were incubated at 37°C on an orbital shaker set at 100 rpm overnight. The following morning, cells were collected and counted. One hundred fifty thousand moDCs were plated in the apical chambers of transwells with 6 µm pore size, and R10++ media was added to the basal chamber with 0; 20; or 200 ng/mL CCL19 (Miltenyi) such that the final concentrations would be 0; 10; and 100 ng/mL CCL19 after diffusing into the apical chamber media. Cells were incubated at 37°C overnight. Cells in the basal chamber were collected using cold PBS (Gibco) containing 5 mM EDTA (Invitrogen). Collected cells were stained with live/dead viability dye and incubated with an antibody to block Fc receptor binding. Antibodies targeting CD11c and CD209 were used to stain cells, and after washing, cells were resuspended in FACS buffer containing counting beads (ThermoFisher). To calculate cell migration, each condition was normalized to control wells where moDCs were plated without transwells, and migration was calculated relative to R848-treated cells incubated without CCL19.

### *In vivo* murine myeloid cell migration

Mice were subcutaneously injected with PBS, R848 (50 µg), and/or CT009 (100 µg) on the upper right back. At 4 hours and 48 hours post-injection, axillary, brachial, and inguinal lymph nodes were harvested from mice and processed using Spleen Dissociation kits (Miltenyi) and the gentleMACS Tissue Dissociator set on program “37C_m_SDK_1.” After dissociation, cells were filtered through 30 µm cell strainers and underwent a cell staining procedure. After washing in PBS, cells were stained with Live/Dead Violet (ThermoFisher) for 20 minutes at 4°C. Cells were then incubated with Fc block (BD Biosciences) for 10 minutes. Cells were then stained with CD11b, Ly6C, F4/80, MHC-I, MHC-II, and CD24 antibodies. After washing, cells were resuspended in FACS buffer containing CountBright beads, and samples were analyzed on the BD Symphony A3 flow cytometer.

### Mouse immunizations

Mice were subcutaneously injected with PBS or Afluria (8 µg/mouse) with/without R848 (50 µg/mouse), CT009 (100 µg/mouse), or Alum (1:1, Adju-phos, Invivogen). Mice received three injections spaced 1 week apart. On day 21, mice were sacrificed and spleens were harvested. Splenocytes were processed to single-cell suspension using Spleen Dissociation Kit (Miltenyi) and gentleMACS Tissue Dissociator (Miltenyi) set to program “37C_m_SDK_1.” After processing, spleens were treated with ACK lysing buffer (Gibco) and resuspended in R10 media. Cells were counted using the Cellaca MX cell counter (Revvity).

### ELISPOT

IFN-γ and IL-5 ELISpot plates (R&D Systems) were blocked with 200 mL R10 media (RPMI-1640 media supplemented with 10% FBS, 100 U/mL penicillin, 100 mg/mL streptomycin, 2 mM L-glutamine, 1 mM sodium pyruvate, and 54 mM beta-mercaptoethanol) for 45 minutes. At the end of blocking, the media was discarded. Splenocytes were seeded at 5E5 cells/well in R10 media and cultured with media alone or media containing 10 µg/mL Afluria Quadrivalent (CSL Seqirus). Plates were incubated at 37°C for 20 hours. After incubation, cells were discarded, and ELISPOTs were developed according to the manufacturer’s instructions. Plates were left to dry overnight at room temperature. The next day, plates were read on the S6 Universal M2 ELISpot plate analyzer, and spots were quantified using the Smart Count function.

### ELISA

Splenocytes were seeded at 5E5 cells/well in 96-well plates and cultured with R10 media alone or R10 media containing 10 µg/mL of Afluria. Plates were incubated at 37°C for 72 hours. Following incubation, plates were spun at 400 x g for 5 minutes, and cell culture supernatants were transferred to 96-well non-tissue culture-treated plates and stored at −80°C until ready for use. Cytokine secretion was measured by IL-5 or IFN-γ ELISA kits from Invitrogen according to the manufacturer’s protocol. Supernatants were thawed at room temperature and diluted 1:20 (IFNγ) or 1:5 (IL-5) in assay buffer and incubated at 4°C overnight on a rocking platform. The following morning, plates were developed according to the manufacturer’s protocol.

### Hemagglutination (HA) and Hemagglutination Inhibition (HAI) assay

All steps in the HA and HAI assay, including washing of the red blood cells (RBCs), were performed in accordance with the World Health Organization guidelines (World Health Organization, 2011). Guinea pig RBCs preserved in Alsever’s solution were washed four times in phosphate-buffered saline (PBS; pH 7.4) by centrifugation at 300 x g for 10 min at room temperature. After the final wash, the pellet was resuspended in 10 mL PBS to generate a 10% stock solution. This stock was kept on ice and diluted to a 0.75% working concentration immediately prior to use.

Prior to performing the HAI assay, mouse sera were treated with receptor destroying enzyme (RDE) (Denka Seiken) for 20 hours at 37°C. RDE was then inactivated at 56°C for 1 hour, and sera were diluted to 10-fold using 0.9% sterile NaCl. After RDE treatment, samples were tested for nonspecific agglutination based on WHO guidelines.

RDE-treated sera were serially diluted twofold in PBS across 96-well round-bottom plates. Afluria (28.8 ng/mL in PBS, 4 HA units) was added to all wells, and samples were incubated for 30 minutes at room temperature. Following incubation, an equal volume of 0.75% guinea pig RBCs was added to all wells, and samples were incubated for 1 hour at room temperature. Hemagglutination inhibition titers were determined as the reciprocal of the highest serum dilution showing complete inhibition, documented using digital imaging.

### PR8 influenza infections

Influenza virus (H1N1) strain A/PR/8/34 (PR8) was purchased from ATCC and propagated in MDCK cells at 37°C for 2 days. For immunizations, the virus was heat-inactivated at 56°C for 1 hour and then quantified by hemagglutinin (HA) ELISA. A total of three experiments were run for monitoring clinical scores and lethality with eight mice per group. A total of two experiments were run for assessing T and B cell readouts with five mice per group. Female BALB/c mice were immunized on day −28 subcutaneously with heat-inactivated virus (IAV) equivalent to 8 ng HA/mouse alone or in combination with R848 (50 µg) and/or CT009 (100 µg) or with MF59 (Addavax, Invivogen 1:1 ratio). Mice received an additional injection 2 weeks later on day −14. On Day 0, mice were challenged intranasally with 1,000 plaque-forming units (PFU) of live PR8 virus. Mice were monitored for body weight changes and scored on a scale of 0 (best) to 3 (worst) for changes in fur coat appearance, mobility, breathing, and conjunctivitis. Four days after challenging a cohort of mice in each group, mice were sacrificed for analysis of immune responses. Antibodies against HA were quantified via ELISA. T cell responses were assessed via nucleoprotein tetramer staining and via IFNγ production after 72 hour restimulation of splenocytes with IAV.

### NHP immunizations

The NHP study was conducted in compliance with all relevant local, state, and federal regulations and was approved by the Institutional Animal Care and Use Committee (IACUC) of BIOQUAL Inc. (Rockville, MD). The study was performed by BIOQUAL (Rockville, MD). A total of 25 cynomolgus macaques were divided into five groups each containing five animals (2 females and 3 males). Each group was immunized subcutaneously with either: Afluria alone (30 ug), Afluria + R848 (180 ug), or Afluria + R848 +CT009 at three different doses (100 ug, 300 ug, or 800 ug). Animals received a second dose 28 days later. Body weight, temperature, and clinical observations were monitored weekly until study end at Day 56. Serum was collected for analysis of antibody titers via HAI assay on Day 0 (pre-dose), Day 28 (pre-dose), and Day 56. PBMCs were collected on Day 0, Day 21, and Day 56 for T cell analysis by ELISpot. At the conclusion of the study, animals were returned to the study pool to be used for future studies by other clients.

HAI assay was performed by BIOQUAL according to BIOQUAL standard procedures. The serum samples were treated with RDE by diluting one part serum with three parts enzyme and incubated overnight in a 37°C water bath. The enzyme was inactivated by a 30 minute incubation at 56°C followed by the addition of six parts PBS for a final dilution of 1:10. The assays were performed in V-bottom 96-well microtiter plates, using 4 HA units of virus (HAU) and 0.5% turkey red blood cells. The reference antiserum for each strain was included as a positive control on every assay plate. Each plate also included a back-titration to confirm the antigen dose (4 HAU/25 µL), as well as a negative control sample. The HAI titer was determined as the highest dilution of serum resulting in complete inhibition of hemagglutination.

IFNγ ELISpot was performed by the Immunology Core at Washington National Primate Research Center (Seattle, WA). IFNγ/IL-4 Double-Color Enzymatic ELISpot assays were performed as per CTL Immunospot (ImmunoSpot, OH, USA) instructions. Briefly, cryopreserved aliquots of NHP PBMC were thawed and then resuspended in serum-free CTL-Test Medium (ImmunoSpot, OH, USA) supplemented with 2 mmol/L L-Glutamine (Gibco, NY, USA) at 7.5e5-1e6 PBMC/mL. PBMCs were plated to 96-well high-protein-binding PVDF filter plates as per kit instructions at 200 µL per well and stimulated in a humidified incubator at 5% CO2, 37°C with 10 µg/mL Afluria Quadrivalent (-), media-only negative controls, and positive controls at 500 ng/mL phorbol 12-myristate 13-acetate (Sigma-Aldrich, MO, USA) and 1 µg/mL ionomycin (Sigma-Aldrich, MO, USA). Plates were developed after 48 hours stimulation and then air-dried in a running laminar flow hood. Plates were scanned, counted, and quality-controlled via S6 Ultimate M2 ImmunoSpot analyzer with ImmunoSpot Pro DC Suite v7 software (ImmunoSpot, OH, USA).

## SUPPLEMENTAL MATERIAL

Supplemental Figures mbio.02343-25-s0001.pdf Figures S1 to S3.

ASM does not own the copyrights to Supplemental Material that may be linked to, or accessed through, an article. The authors have granted ASM a non-exclusive, world-wide license to publish the Supplemental Material files. Please contact the corresponding author directly for reuse.
