# Supplementary material for: An NLRP3-stimulatory adjuvant improves the immunogenicity of influenza virus vaccines in mice and non-human primates
Source: mBio. 2025 Sep 8;16(10):e02343-25. doi: 10.1128/mbio.02343-25 (PMC12505977; doi:10.1128/mbio.02343-25)
Supplement: Supplemental Figures — Figures S1 to S3. [file mbio.02343-25-s0001.pdf]

Supplemental Figures

A

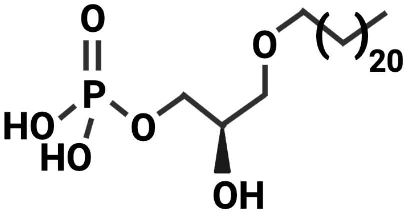

B

CT009 Lot	Size Dv(50) µm (uniformity)	pH	Osmolality (mOsm/kg)
1	9.79 (0.474)	6.66	334 ± 3
2	18.3 (0.498)	6.62	345 ± 2
3	15.7 (0.607)	6.62	347 ± 1

C

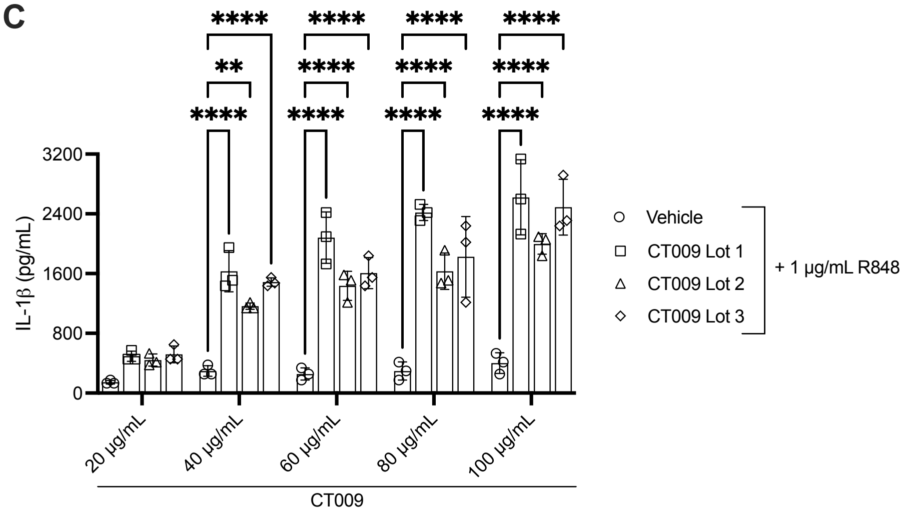

D

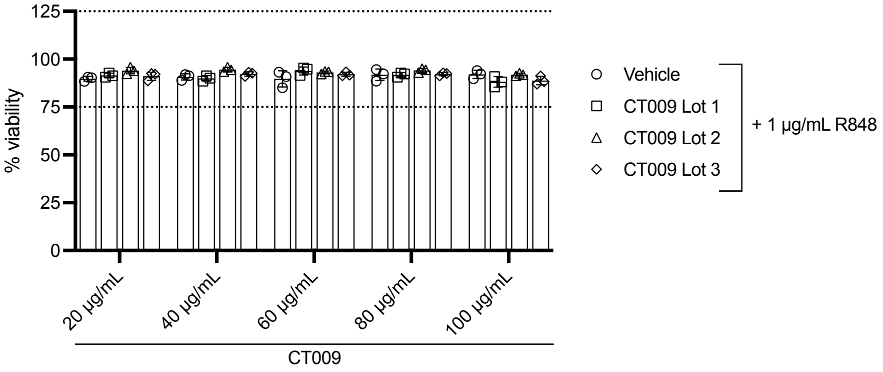

Supplemental Figure 1. Synthesis of hyperactivating lipid CT009. (A) Drawing depicts chemical structure of CT009. (B) Three lots of CT009 were manufactured and formulated. Each lot was characterized for its particulate size (median volume distribution), uniformity, pH, and osmolality. (C) THP-1 Null2 cells were treated with 1 µg/mL R848 and various concentrations of CT009 as labeled. Two days later, IL-1β was measured in the cell culture supernatant. (D) Lactate dehydrogenase release was quantified as a measure of viability for supernatants. Each symbol represents one technical replicate. Statistical significance was determined using two-way ANOVA followed by Tukey's multiple comparisons test. **p<0.01; ****p<0.0001

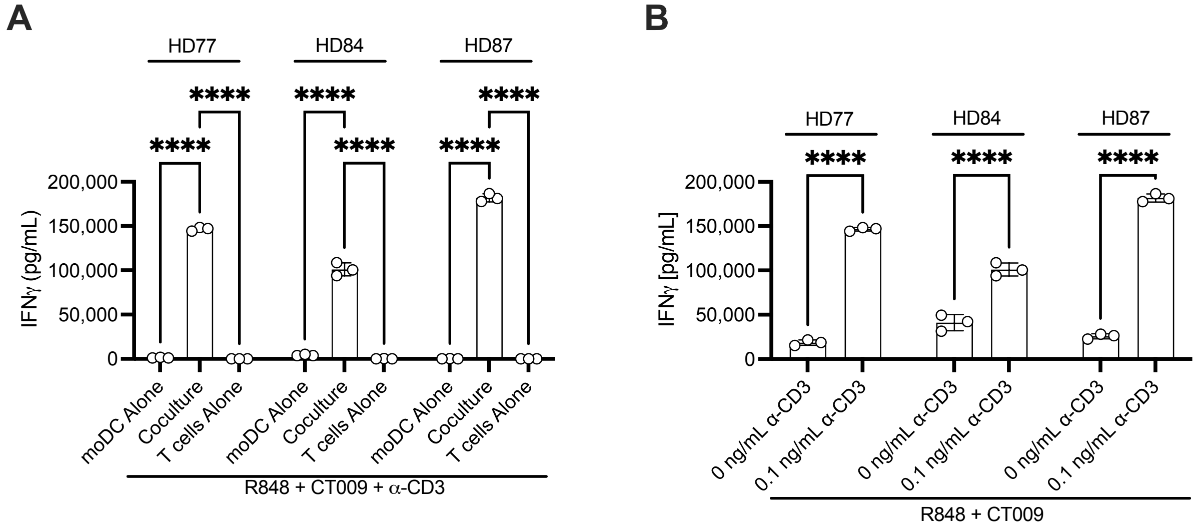

Supplemental Figure 2. moDC-T cell culture responses require the interaction of both cell types and a T cell receptor stimulus. (A) Control conditions to cocultures were plated with hyperactivating chemicals but lacked either moDC or CD4 T cells. (B) In another set of controls, cocultures were plated with 1 μg/mL R848 and 41.3 μM CT009 but lacked anti-CD3 antibody. HD = healthy donor. Each symbol represents one technical replicate. Two-way ANOVA followed by Tukey's multiple comparisons test was used to determine significant differences between treatment groups. ****$p<0.0001$

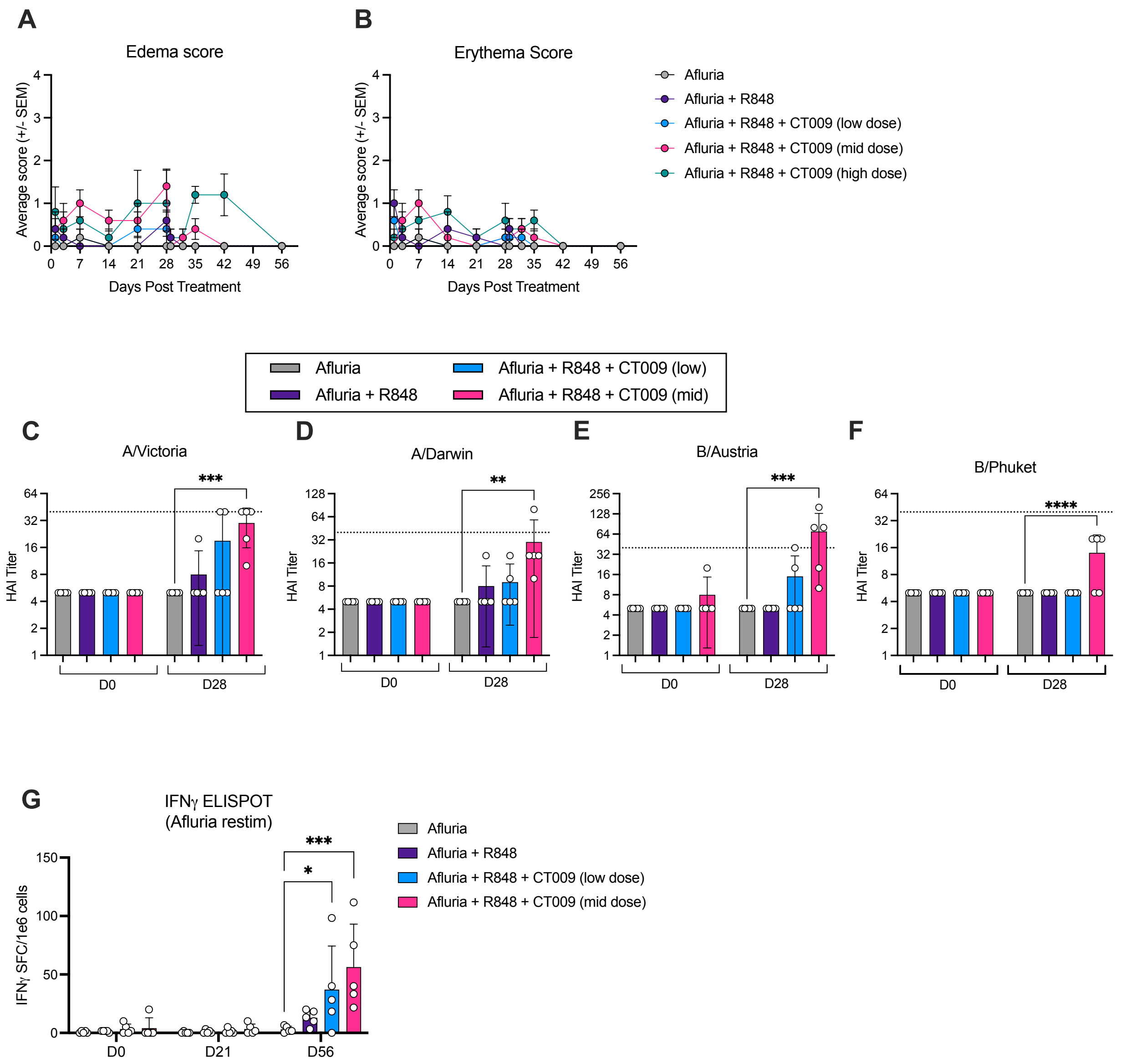

Supplemental Figure 3. Additional NHP reactogenicity and immunogenicity to vaccination data. Animals were scored weekly for (A) edema and (B) erythema at injection sites. (C-F) HAI titers against each influenza strain in Afluria were measured from serum on days 0 and 28. (G) Antigen-specific T cell responses were measured by IFNγ ELISPOT from PBMCs taken on days 0, 21, and 56. Reported responses have unstimulated background signals subtracted. Means and SD are shown. Each symbol in bar graphs represents one animal. Significance was determined by Two-way ANOVA with Dunnett's multiple comparisons test to Afluria. **p<0.01; ***p<0.001; ****p<0.0001
